# A mechanistic investigation of the oxygen fixation hypothesis and oxygen enhancement ratio

**DOI:** 10.1088/2057-1976/1/4/045209

**Published:** 2015-12-04

**Authors:** David Robert Grimes, Mike Partridge

**Affiliations:** Cancer Research UK/MRC Oxford Institute for Radiation Oncology, Gray Laboratory, University of Oxford, Old Road Campus Research Building, Off Roosevelt Drive, Oxford OX3 7DQ, UK

**Keywords:** radiotherapy, oxygen, oxygen effect, radiation damage

## Abstract

The presence of oxygen in tumours has substantial impact on treatment outcome; relative to anoxic regions, well-oxygenated cells respond better to radiotherapy by a factor 2.5–3. This increased radio-response is known as the oxygen enhancement ratio. The oxygen effect is most commonly explained by the oxygen fixation hypothesis, which postulates that radical-induced DNA damage can be permanently ‘fixed’ by molecular oxygen, rendering DNA damage irreparable. While this oxygen effect is important in both existing therapy and for future modalities such a radiation dose-painting, the majority of existing mathematical models for oxygen enhancement are empirical rather than based on the underlying physics and radiochemistry. Here we propose a model of oxygen-enhanced damage from physical first principles, investigating factors that might influence the cell kill. This is fitted to a range of experimental oxygen curves from literature and shown to describe them well, yielding a single robust term for oxygen interaction obtained. The model also reveals a small thermal dependency exists but that this is unlikely to be exploitable.

## 1. Introduction

Oxygen is of vital importance in radiotherapy response [1]. Since the 1950s [2], it has been repeatedly demonstrated that tumours with better oxygenation respond markedly better to radiotherapy then those with extensive hypoxia [3-5]. The presence of molecular oxygen significantly modifies the effectiveness of radiotherapy; relative to anoxia, well oxygenated tumours respond better by a factor of 2.5–3. This boosting fraction is referred to as the oxygen enhancement ratio (OER). Half maximum radio-sensitivity occurs somewhere around an oxygen partial pressure of 3 mmHg, with maximum OER typically achieved at partial pressures *p* > 20 mmHg, with subsequent increases not significantly modifying the curve [1, 6]. Experiments performed in cells, yeast and bacteria conform to the same general OER curve, which rises and quickly saturates [1], obeying a roughly hyperbolic relationship with oxygen tension [1, 7, 8]. In radiotherapy, OER is vitally important, potentially boosting treatment effect up to three-fold. It is of current research interest also, particularly in dose delivery to hypoxic sub-volumes of tumours using PET guided IMRT boose treatments [9]. First principles analysis of radiation damage are rare [10] and to our knowledge none to date deal specifically with oxygen mediated damage. To estimate dose dynamics, improved understanding of the physical basis of the OER would add useful information over current empirical models.

**Figure 1:**
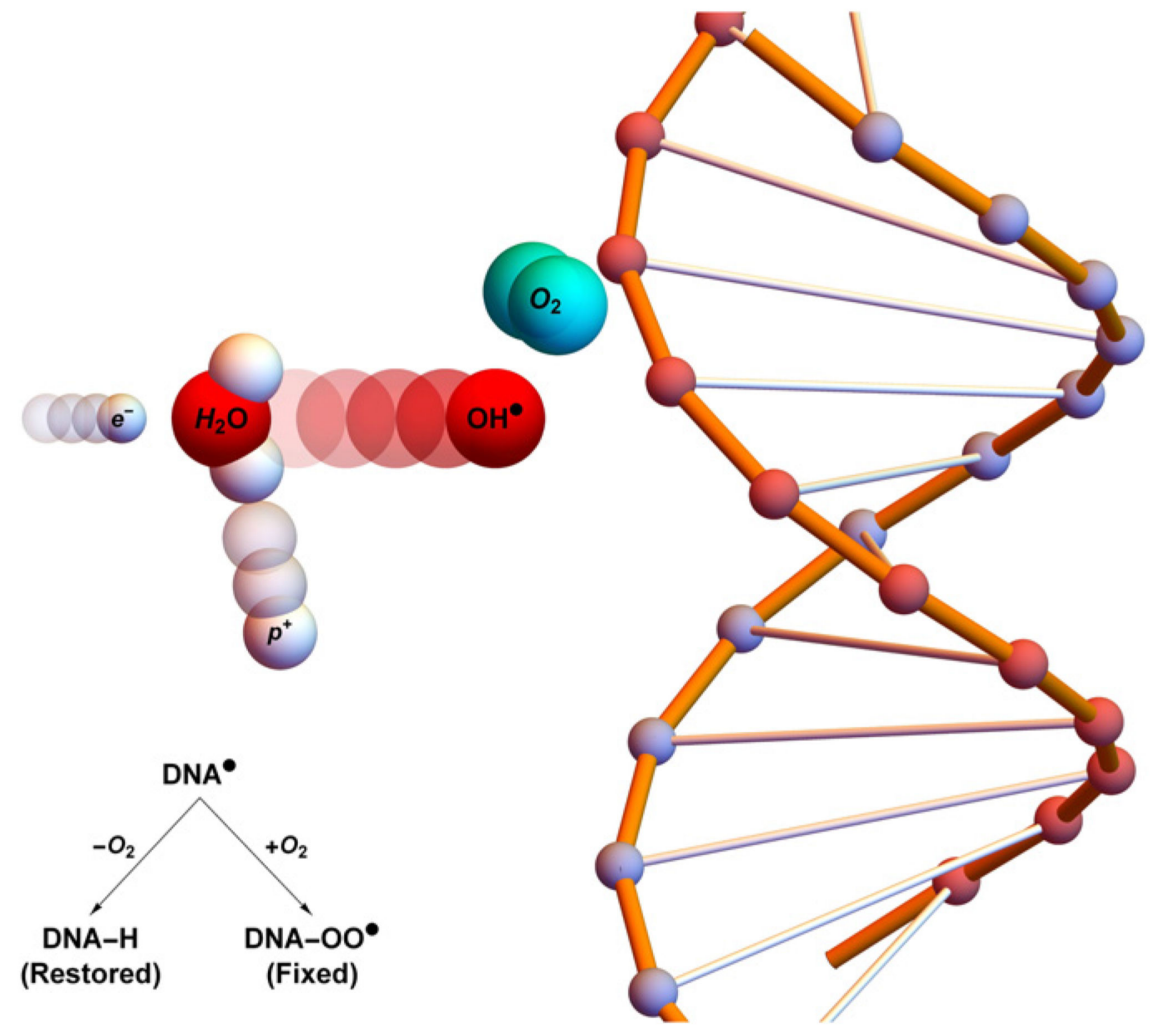
Oxygen fixation hypothesis—a high energy electron created by an x-ray photon (*e*^−^) impinges upon a water molecule, liberating a proton (*p*^+^) and creating a hydroxyl radical (OH^•^). This reactive molecule then impacts upon DNA, resulting in ionization damage, DNA^•^. This can be readily repaired to its original state (DNA-H), but in the presence of molecular oxygen a peroxy radical is formed (DNA-OO^•^), ‘fixing’ damage into a permanent irreparable state.

For the oxygen enhancement effect to be observed, molecular oxygen must be present before irradiation or within microseconds of exposure [1, 11]. No increase in OER occurs if oxygen is added beyond this threshold. The radiochemical rationale for this is known as the oxygen fixation hypothesis (OFH) [1, 7, 12]. OFH postulates that most DNA can be repaired after radical damage, but that repair is more difficult or impossible when caused by the product of a radical and an oxygen molecule. When an incoming high energy photon interacts with biological matter, it can cause damage in several ways. It can directly interact with DNA, causing an ionization event. More commonly, it can interact with other organic matter such as water, producing high energy electrons. These ionizing electrons react with water to create highly reactive hydroxyl radicals (R^•^) which in turn cause DNA base damage. In general, this kind of radical damage is readily chemically repaired. However, when these radicals encounter molecular oxygen they form a peroxyl radical, RO_2_^•^. This is much more damaging, difficult or impossible to for the cell to repair. In essence, damage produced by free-radicals can be restored under hypoxia but is ‘fixed’ (made permanent and irreparable) when molecular oxygen is present [12, 13], as illustrated in figure 1.

While the OFH is commonly accepted as the mechanism behind the oxygen effect [1, 12], there has been relatively little work on the fundamental physics of the oxygen interaction, nor of the physical parameters modifying the extent of this effect. OER is nonlinear with concentration of molecular oxygen, displaying an abrupt saturation curve, and models describing this would be useful. For future treatment modalities such as hypoxic dose-painting, quantifying of these parameters is of high importance. In this work, we derive a model for OER from physical first principles, fitting it to a number of classic OER experiments [14-16]. We also investigate modifiable parameters, and whether these can be manipulated to boost the oxygen effect, and sensitivity of the OER to these variables.

## 2. Model derivation

If the OFH holds, molecular oxygen rapidly interacts with ionized DNA within a very short time frame. This is a rare event, proportional to oxygen concentration, itself proportional to oxygen partial pressure *p* [17]. Poisson statistics can be employed to determine the probability *f* that ionized DNA interacts with at least one oxygen molecular pair, given by

(1) $$f=1-e^{-\varphi \left(p\right)},$$

where *φ* is a function of oxygen partial pressure. Defining *ω*(*p*) as the number of oxygen molecules contained in an interaction volume V_I_ and *ν* as probability that a given oxygen molecule at interacts with ionized DNA, we write

(2) φ(p)=ν(ω(p)).

We may quantify *ω* in terms of *p* from first principles through some manipulation [17]. If *C* specifies the volume of oxygen gas per unit tumour mass, this can be related to oxygen partial pressure *p* by $C=\frac{p}{\Omega }$, where Ω is a constant arising from Henry’s law and related to the density of the tumour (taken as that of water) and oxygen gas. Henry’s law exhibits thermal dependence, so we initially take this at human body temperature, yielding Ω = 3.0318 × 10^7^ mmHg kg m^−3^ as previously derived [17]. For *C* (*p*), the fraction of oxygen to tumour mass is the product of the oxygen gas density ρ_O_2__ with *C* (*p*). Thus, the number of oxygen molecules per unit tumour mass can be obtained by dividing through the mass of an oxygen molecule *m*_O_2__. Finally, the number of molecules per unit tumour volume is found by multiplying through by unit tumour density *ρ*_T_. Across the interaction volume *V*_I_, the number of O_2_ molecules is given by

(3) $$\omega \left(p\right)=\left(\frac{V_{I}\rho_{O_{2}}\rho_{T}}{\Omega m_{O_{2}}}\right)p.$$

The interaction probability for a single oxygen molecule impinging upon ionized DNA, *ν*, can be derived from first principles. From kinetic theory, molecules are in a constant state of flux, undergoing multiple collisions. We define *ε* as the probability of an interaction with ionized DNA per collision for a single molecule and *η* as the number of collisions a molecule undergoes in a given period of time. Thus we may state that the chance of at least one interaction is given by

(4) $$\nu =\left(1-{\left(1-\varepsilon \right)}^{\eta }\right).$$

This can be specified further; the thermal velocity of oxygen molecules is given by the kinetic theory as

(5) $$\upsilon_{T}=\sqrt{\frac{8k_{B}T}{\pi m_{O_{2}}}},$$

where *k*_B_ is the Stefan–Boltzman constant. The mean-free path of oxygen in a liquid, *l_f_*, is related to both thermal velocity *υ*_T_ and the oxygen diffusion constant *D*, given by

(6) $$l_{f}=\frac{3D}{\upsilon_{T}}.$$

The number of collisions per unit time is the ratio of *υ*_T_/*l_f_*. OFH predicts that DNA damage can be rapidly repaired in the absence of oxygen, so for fixation to occur oxygen must impinge close to the site of ionization within the experimentally derived time-frame *τ_E_*. The maximum number of collisions a single molecule of oxygen undergoes in the span *τ_E_* is

(7) $$\eta_{r}=\frac{\upsilon_{T}\tau_{E}}{l_{f}}=\frac{\upsilon_{T}^{2}\tau_{E}}{3D}.$$

The identity in equation (7) gives an upper limit for collision events, but there is experimental evidence suggesting the chances of interaction events decay exponentially over the excitation window, with a half-life of approximately 500 *μ*s [1, 11]. This might be due to the probabilistic nature of ionization life-time, and we can factor this to obtain better collision estimates. The decay constant can be calculated from the half-life and the decay curve integrated over the time interval 0 ⩽ *t* ⩽ *τ_E_* to yield a more robust estimate for the number of collision events, given by

(8) $$\eta_{e}=\frac{\upsilon_{T}}{l_{f}\lambda }\left(1-e^{-\lambda \tau_{E}}\right).$$

We can thus write the total interaction probability as

(9) $$\nu =1-{\left(1-\varepsilon \right)}^{\frac{\upsilon_{T}}{l_{f}\lambda }\left(1-e^{-\lambda \tau_{E}}\right)}.$$

The average interaction volume can be derived from statistical mechanics; oxygen molecules have mean-free path of *l_f_*, travelling randomly with its course deflected by collisions. In a window of *τ* we expect *η*_*r*_ collisions, modelled as the expectation value of a random walk in three-dimensions (*d* = 3). The expectation value for an O_2_ molecule from the origin is related to the mean of chi-distribution, given by

(10) $$\langle r\rangle =l_{f}\sqrt{\frac{\eta_{r}}{d}}\left(\frac{\Gamma \left(\frac{d+1}{2}\right)}{\Gamma \left(\frac{d}{2}\right)}\right)=2l_{f}\sqrt{\frac{2\eta_{r}}{3\pi }},$$

where Γ is the gamma function, and the interaction volume is simply a sphere of radius 〈*r*〉, thus

(11) $$V_{I}=\frac{4\pi }{3}{\left(2l_{f}\sqrt{\frac{2\eta_{r}}{3\pi }}\right)}^{3}.$$

These terms combined yield an explicit identity for *φ* of

(12) $$\begin{array}{cc} \varphi & =\frac{\nu \left(\omega \left(p\right)\right)}{p}\\ & =\left(\frac{V_{I}\rho_{O_{2}}\rho_{T}}{\Omega m_{O_{2}}}\right)\left(1-{\left(1-\varepsilon \right)}^{\frac{\upsilon_{T}}{l_{f}\lambda }\left(1-e^{-\lambda \tau_{E}}\right)}\right). \end{array}$$

We can quantify total cell kill as comprising of two terms; one with an oxygen dependence and one without. We define *N*_P_ as the photon flux and *t* as the exposure time. At a given photon energy, we define the fraction of cells without oxygen fixation as *ϕ_D_*, and the fraction killed by radical fixation as *ϕ_O_*. The collision cross-section *σ_E_* is energy-dependent, and readily obtained from NIST tables. Total cell kill *χ*(*p*) is thus given by

(13) $$\chi \left(p\right)=N_{P}\;\sigma_{E}t\left(\phi_{D}+\phi_{O}\left(1-e^{-\varphi p}\right)\right).$$

This theoretically quantifies the increased cell kill due to the oxygen fixation effect. This can be related to maximum OER, typically defined as the relative increase in cell kill under completely oxic conditions relative to cell kill under anoxia at a given dose. To estimate the maximum possible OER, we find the limit of

(14) $${\mathrm{OER}}_{\mathrm{Max}}=\lim_{p\to \infty }\frac{\chi \left(p\right)}{N_{P}t\left(\sigma_{E}\right)\phi_{D}}=1+\frac{\phi_{O}}{\phi_{D}}.$$

Thus, we can establish an identity that relates OER to oxygen partial pressure by

(15) $$\mathrm{OER}\left(p\right)=1+\left(\frac{\phi_{O}}{\phi_{D}}\right)\left(1-e^{-\varphi p}\right).$$

### 2.1. Parameter estimation

Parameter values taken from literature are shown in table 1. Values derived through the equations outlined in this work are shown in table 2.

## 3. Experimental method

OER curves as a function of *p* were taken from literature [14-16]. All values of *p* over the entire spectrum of human physiological oxygen (0 ⩽ *p* ⩽ 160 mmHg) were considered. The data-sets consider spanned a range of energy levels differing by a factor of over 400 (shown in table 3). Equation (15) was fitted independently to each data set, with the MATLAB Nonlinear least squares package to estimate best fit parameters and confidence intervals. All data points from all experiments were pooled and the same analysis performed.

### Estimation of interaction probability

As all parameters are known or measurable for number of O_2_ molecules in the interaction volume *ω*(*p*), then for any experimentally measured *φ*, $\nu =\frac{\varphi }{\omega (p)}$, yielding total probability of interaction between ionized DNA and oxygen. It was possible to obtain literature estimates for all parameters except *ε*, the probability of an interaction between ionized DNA and an oxygen molecule per collision. This is expected to be minute, but can in principle be estimated from measured *φ* by

(16) $$\varepsilon =1-{\left(1-\nu \right)}^{\frac{1}{\eta_{e}}}$$

but caution must be taken to avoid large radical errors.

### Theoretical temperature dependence of *φ*

Of all the parameters in the model, most are fixed constants and cannot be modified. There is one possible exception however; there are two terms in (15) which have an explicit dependence on body temperature *T*. Firstly, the Henry’s law solubility constant for oxygen gas *K* depends on temperature [20], which in turn modifies Ω [17]. This temperature dependence can be described by

(17) $$\Omega \left(T\right)=\left(23.75\rho_{O_{2}}\rho_{T}\kappa_{c}\right)\exp\left(-C\left(\frac{1}{T}-\frac{1}{T_{\Theta }}\right)\right),$$

where *κ_c_* is Henry’s constant value at reference temperature, *T*_Θ_ is the reference temperature and *C* is a constant for the gas. For oxygen, the reference temperature is *T*_Θ_ = 298.15 *K, κ_c_* = 769.23 L atm/mol and *C* = 1700 K [21]. From equation (5) it is clear that oxygen molecules have a well-defined thermal dependence. This in turn means that *l_f_, η_r_, η_e_* and *V*_I_ have readily calculated thermal variation. We may then re-write number of particles in an interaction volume *ω* as a function of temperature by

(18) $$\omega \left(T\right)=\left(\frac{V_{I}\left(T\right)\rho_{O_{2}}\rho_{T}}{\Omega \left(T\right)m_{O_{2}}}\right)p\left(T\right).$$

To use this identity, we need to quantify how O_2_ partial pressure *p*(*T*) is affected by temperature. The interaction volume is contained within a semi-infinite fluid, so we may use the ideal gas law with a non-compressible equation of state equation (*PV* = *Nk*_B_ *T*) assuming the *V*. With re-arrangement and allowing for appropriate conversation between units (133.22 Pa = 1 mmHg), we rewrite the thermal dependence as *p* = *ZT*, where *Z* is a constant given by $Z=\frac{Nk_{B}}{133.22\;V}$. Defining partial pressure at human body temperature as *p_o_*, we derive an expression for the projected change in partial pressure with temperature of

(19) $$p\left(T\right)=p_{o}\left(\mathrm{ZT}\right),$$

where the constant *Z* can be ascertained to have a value of *Z* = 3.22 × 10^−3^ K^−1^. The interaction probability *ν* has a small theoretical thermal dependence which can be calculated from equation (9).

### Investigation of energy dependence of OER

The wide range of energies used in the experimental data (shown in table 3 was used to investigate whether there is a significant relationship between photon energy and $\frac{\phi_{O}}{\phi_{D}}$. For each data set, the mean value and standard deviation were calculated from fitting and an ANOVA test performed. A similar ANOVA analysis was performed for *φ*.

## 4. Results

### Comparison of model with data

**Figure 2:**
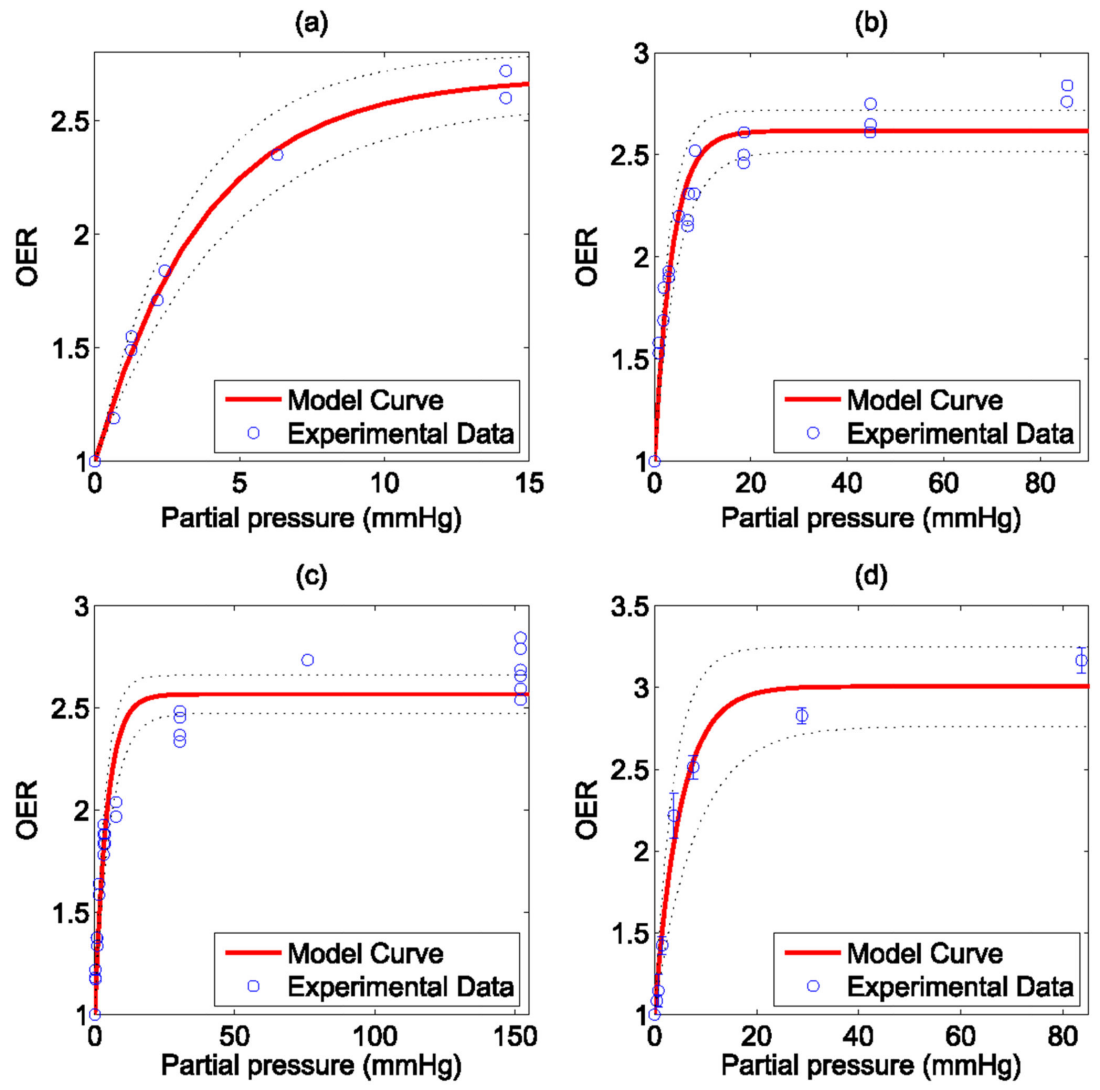
Model fit to historical OER data from (a) Koch *et al* (b) Whillians and Hunt (c) Ling *et al* (a) (d) Ling *et al* (b). 95% confidence intervals shown by dotted black lines.

**Figure 3:**
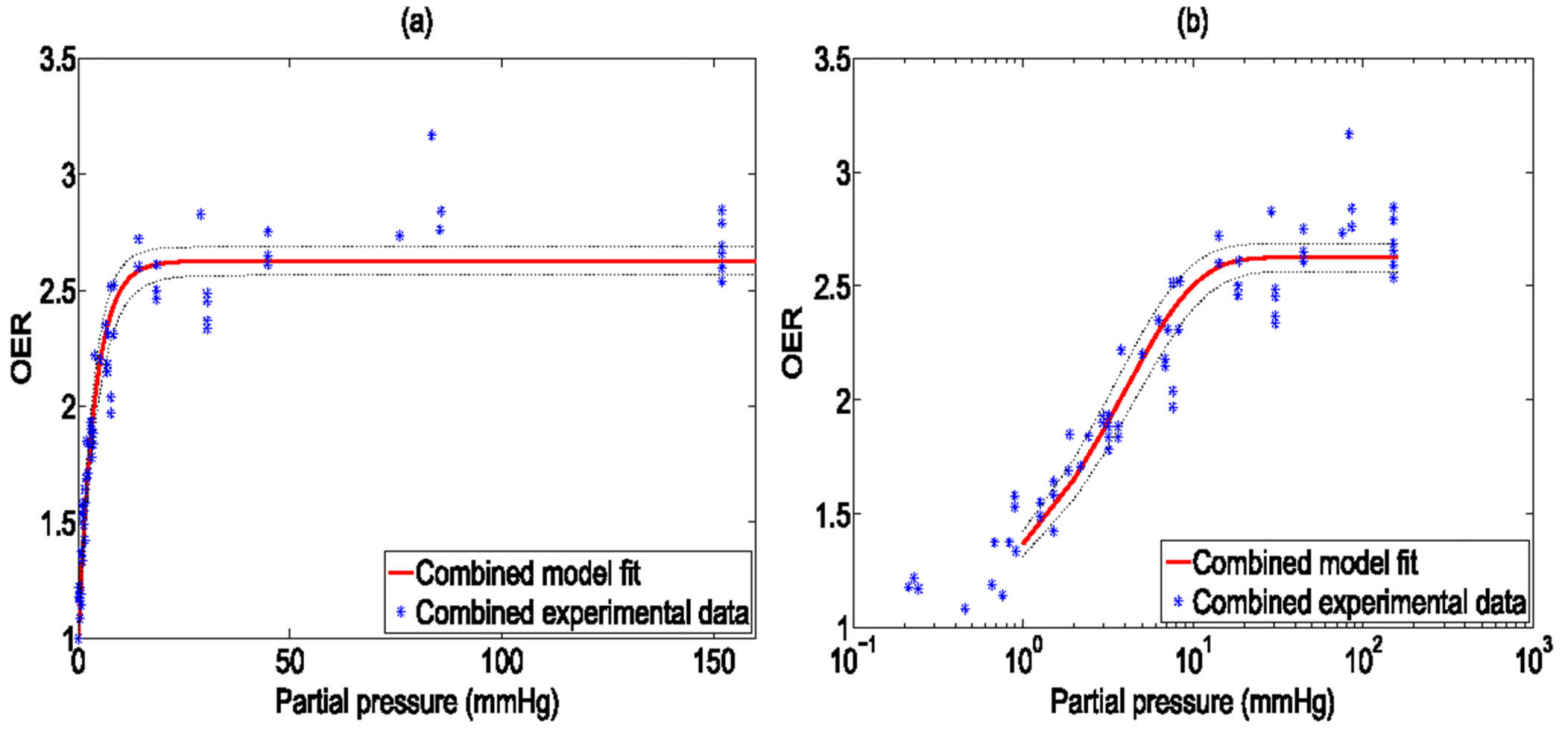
Model fit from combined data sets shown with (a) standard *x*-axis (b) logarithmic *X*-axis for clarity. 95% confidence intervals shown by dotted black lines.

The model was fitted to the experimental data and goodness of fit calculated and 95% confidence intervals using a nonlinear least squares algorithm, illustrated in figure 2. All points were then pooled and the same analysis was performed, illustrated in figure 3. Results and estimated values are shown in table 4, yielding good fits to experimental data.

### Estimation of interaction probability

The estimated interaction probability between ionized DNA and an oxygen molecule was estimated as *ν* = 2.14 ± 0.32 × 10^−6^ with a value of *ν* ≈ 2.28 × 10^−6^ for the combined set. Estimates of *ε* were obtained from equation (16) with high precision calculations employed in Mathematica to prevent large errors being introduced. This yielded *ε* = 9.24 ± 1.40 × 10^−17^, and *ε* = 9.85 × 10^−17^ from the combined set. Results for *ν* are robust, but should be interpreted carefully for *ε*; in equation (15), the exponent was taken as $\frac{1}{\eta_{e}}$ to reflect the observed decrease in collision events with time. There is inherent uncertainty in this; for example, if $\frac{1}{\eta_{r}}$ (the maximum number of collisions) is used instead (differing by a factor of ≈3), then there is in estimate value by three orders of magnitude, *ε* = 6.67 × 10^−20^. Small errors here amplify, but across all data sets *ν* remains consistent.

### Theoretical thermal dependence

**Figure 4:**
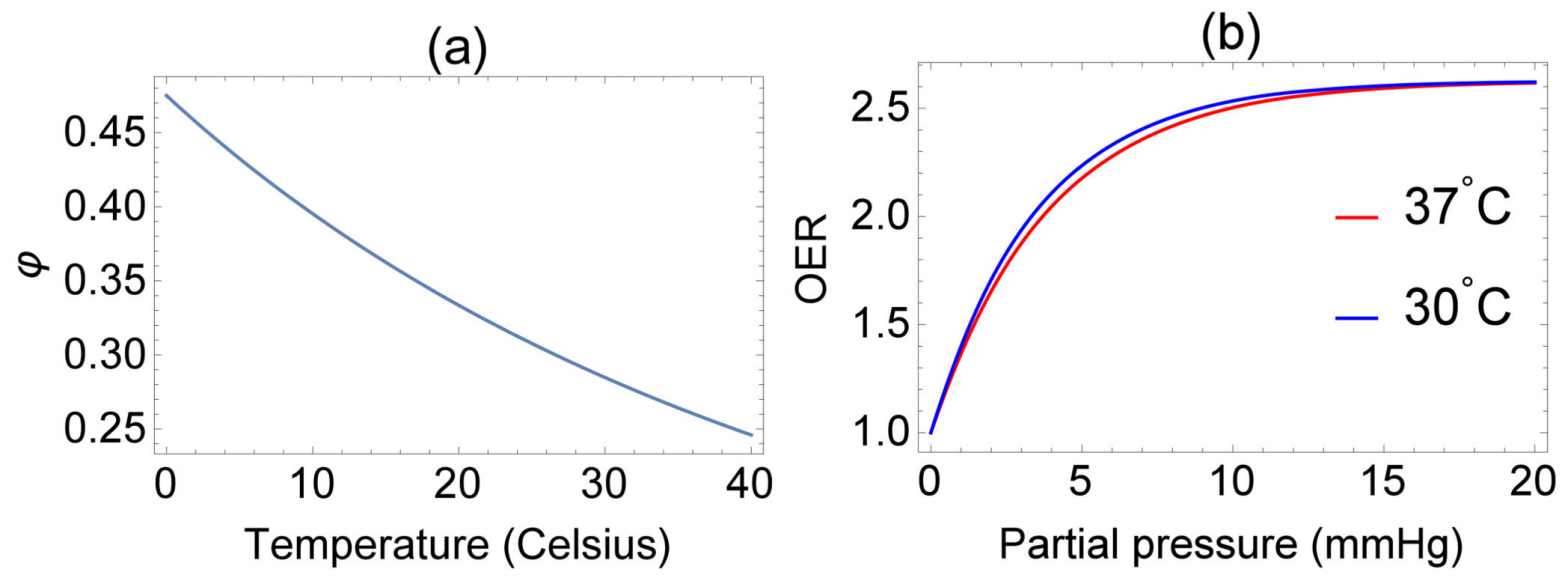
(a) Temperature dependence of *φ* over a 40 K range (b) projected OER curves at different temperatures.

The projected oxygen enhancement effect has a quasilinear thermal dependence, with decreasing temperature resulting in increased values of the oxygen saturation term *φ*. This is illustrated in figure 4(a). In principle, this means increased OER at lower partial pressures as illustrated by figure 4(b). Table 5 shows the variation across temperature ranges, assuming *φ* = 0.2567 mmHg^−1^ at human body temperature, and the fractional change in *φ*(*T*) with temperature. Decreasing treatment site temperature to 32 °C would raise the saturation constant by about 6%. The clinical relevance of this is explored in the discussion.

### Investigation of energy dependence of OER(*p*)

Over all the energy ranges in the data-sets, the estimated values of $\frac{\phi_{O}}{\phi_{E}}$ were analysed with an ANOVA statistical test to examine whether there was any significant energy dependencies. Results of this analysis indicated that $\frac{\phi_{O}}{\phi_{E}}$ at a photon energy of 280 kVp yielded a significantly significant (*p* = 0.004) different from the other data sets. The implications of this are outlined in the discussion. A similar analysis for *φ* yielded no statistically significant energy dependence.

## 5. Discussion

The first principles model fits all observed data sets well, with co-efficient of determinations between 0.92 ⩽ *R*^2^ ⩽ 0.99 and remarkably consistent model fit parameters, despite the substantial variation in energy ranges, cell type and experimental techniques used [6, 14-16]. This suggests the underlying theory is sufficiently robust to model the behaviour of a range of published experimental data from first principles. The model explains the mechanism behind the observed rapid saturation of the oxygen effect to a maximum; previously, this had been described by a hyperbolic curve as a phenomenological device [7] without reference to the underlying physics. This work derives all parameters from first principles, suggesting the observed curve arises from the Poisson-like saturation point of oxygen molecules in an interaction volume. Most physical parameters could be estimated directly from theory or literature, with the exception of *ν* (total interaction probability for an oxygen molecule with ionized DNA). This could be readily estimated from fitting, yielding values of 2.14 ± 0.32 × 10^−6^. From this, estimates of *ε* (the per-collision probability of ionized DNA interacting with a given oxygen molecule) were also possible, with results of 9.24 ± 1.40 × 10^−17^ across all data sets. These magnitudes are in line with prediction, though estimates of *ε* must be cautiously interpreted.

The model also explains features of the data—typically, OER curves achieve half-maximum increase values around *p* ≈ 3 mmHg [1], saturating by 20 mmHg. Using our experimentally derived values for *φ*, it is possible to examine this more precisely. At a partial pressure *p*, the probability *P*(*p*) of at least one interaction between the oxygen molecules and an ionized DNA molecule is given by

(20) $$P\left(p\right)=\sum_{k=1}^{k=\infty }\frac{{\left(\varphi p\right)}^{k}e^{-\varphi p}}{k!}.$$

The interaction probability is given in table 6, and as expected the probability of an oxygen-radical event at 3 mmHg is close to half. By 20 mmHg, interaction probability is above 99%, in agreement with literature interaction.

**Figure 5:**
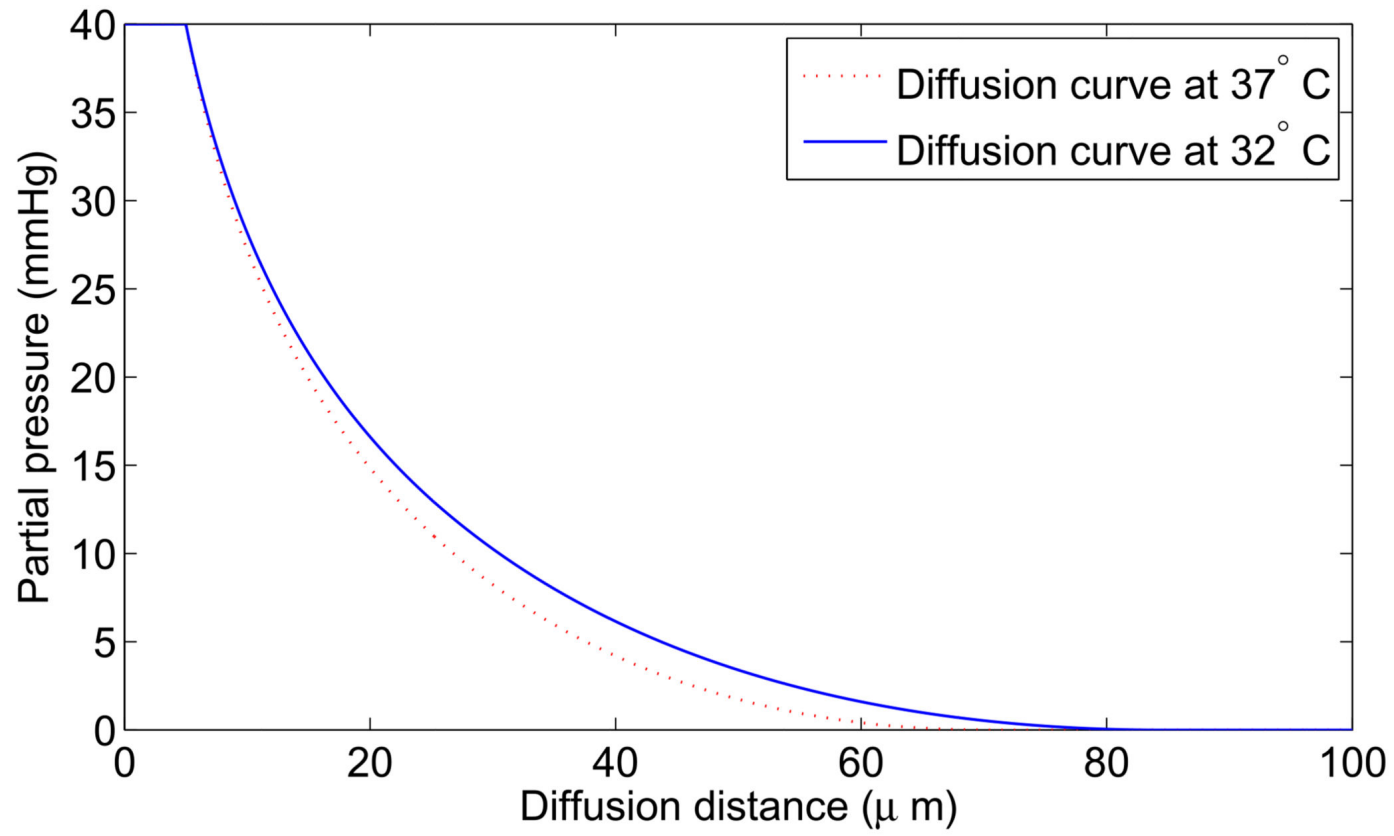
Diffusion from a vessel of 5 *μ*m radius at venous pressure (40 mmHg). At human body temperature, this example has oxygen consumption rate *a* = 5 × 10^−7^ m^3^ kg^−1^ s^−1^ with maximum diffusion distance 70.16 *μ*m. Under low temperature (32 °C), Ω(*T*) = 2.7714 × 10^7^ mmHg kg m^−3^ and *a* ≈ 3.5 × 10^−7^ m^3^ kg^−1^ s^−1^, increasing diffusion distance to 84.22 *μ*m, potentially increasing available oxygen for radiotherapy. Curves calculated from simple Krogh-type model as previously outlined [19].

The thermal dependence of *φ* is an interesting consequence of theory; induced hypothermia is already available in clinical practice, used to boost patient survival by reducing injury to tissue from lack of blood-flow, typically following a cardio-vascular accident or head-trauma [22-24]. Patients are typically cooled to 32 °C–34 °C in these situations, theoretically increasing *φ* by 6% as per table 5. This effect is not particularly substantial—at 32 °C, the maximum increase in OER would be 1.8%, with an average increase of just 1% between 0 ⩽ *p* ⩽ 20mmHg, rendering it unlikely to be clinically exploitable. Lower temperatures do however raise another interesting theoretical possibility; metabolic demand decreases with temperature, with each 1 K drop corresponding to a reduction in oxygen demand between 5% and 7% [25]. Some animal experiments [27] suggest oxygen supply is relatively unaffected by hypothermia, in which case a decreased oxygen consumption would imply increased oxygen diffusion [17, 19] as illustrated in figure 5. In reality, the situation is likely much more complicated. To the authors knowledge, there have been no studies of radiotherapy under low temperatures, but mild hyperthermia (as opposed to hypothermia) has been studied and shown to improve radio-sensitization [27, 28] and nano-particle delivery [29]. There is some evidence that hyperthermia increases blood-flow [30], and others suggesting that tumour oxygenation increases with low thermal doses but decreases at higher temperatures [31]. Higher temperatures themselves (>42.5 °C) are cytotoxic, increasing radiosensitization due to inhibited repair of DNA lesions [27, 28] rather than any oxygen effect. While the model here predicts a small increase in OER with decreasing temperature, it is unlikely that this effect is clinically exploitable. The question of whether lower temperatures would improve oxygen diffusion is an interesting one, but beyond the scope of this work.

It is also important to note OFH only covers chemical repair rather than biological and enzymatic DNA repair processes that may occur *in situ*. While chemical repair is completely independent to the biology of the target, biological repair pathways can differ between species—at least one study [32] has demonstrated that the maximum insignificant dose (the maximum dose that does not reduce survival) demonstrates strain dependence between different types of *E-coli*. In this work, we confine our attention solely to chemical fixation and how oxygen moderates this effect.

As OER is relative, any explicit energy dependence should cancel out as shown in equation (15). Statistical tests for fitted *φ* in this work indicate no energy dependence, in line with prediction. An ANOVA analysis for $\frac{\phi_{O}}{\phi_{D}}$, the fraction of damage events in the presence of oxygen over the fraction of damage events under anoxia, was also considered. For three of the four sets there was no significant variation in this fraction with energy, but for the data set with a peak of 280 kV there was a statistically significant difference. The reason for this is unclear; it might indicate a real phenomenon or systematic measurement error. The analysis shown in table 4 reveals the standard error of this data-set is the greatest of all considered, and the set itself contains the least number of points of all those considered, possibly lending itself to fitting error. No obvious trend for $\frac{\phi_{O}}{\phi_{D}}$ was observed across the other data sets. Further experimental data would be required to determine whether any true energy dependency exists. In all cases, the model described the underlying data well. We conclude that the theory derived here is readily applicable for quantifying the oxygen effect from x-ray photons over a range of energies. It should be noted our model is unlikely to describe the case of high linear-energy-transfer (LET) particles, as the greater damage density in DNA by such particles is generally not reparable and thus oxygen fixation does not apply [1]. By contrast, OER remains a vitally important component in photon and low LET therapy, which constitutes the majority of radiotherapy modalities.

## 6. Conclusion

The results in this work support the OFH that free radical induced DNA damage is magnified in the presence of molecular oxygen, and offer a mechanistic explanation of the likely parameters that influence oxygen effect. The model presented agrees well with a wide range of classic oxygen effect experiments, and explains why the characteristic oxygen enhancement curves has its distinctive shape. The work presented here also explores the physical constants involved in the oxygen effect from first principles. While most of these parameters are fixed natural constants, there is a small theoretical thermal effect but it is unlikely this can be exploited in a clinical setting.

## Figures and Tables

**Table 1:** Parameter values from literature.

Parameter	Value
Oxygen gas density—*ρ*_*O*2_ [17]	1.331 m^3^ kg^−1^
Tumour density—*ρ*_T_^a^	1000 m^3^ kg^−1^
Mass of O_2_ molecule—*m*_O2_	5.314 × 10^−26^ kg
Diffusion constant of O_2_ in water—*D*[17–19]	2 × 10^−9^ m^2^ s^−1^
Maximum repair window—*τ_E_*[1]	2 ms
Half-maximum oxygen effect window—*t*_0.5_ [1]	500 *μ*s

a Taken to be approximately same as water.

**Table 2:** Parameter values from derived identities.

Parameter	Value
Thermal velocity O_2_—*ν*_T_	452.890 ms^−1^
Mean-free path O_2_—*l_f_*	1.325 × 10^−11^ m
Time-decay constant—λ	1.386 × 10^3^ s^−1^
Maximum collision—*η_r_*	6.840 × 10^10^
Likely collision—*η_e_*	2.310 × 10^10^
Interaction volume—*V*_I_	1.363 × 10^−16^ m^3^
Number O_2_ molecules per *V*_I_—*ω*(*p*)	(1.126 × 10^5^)*p*

**Table 3:** Photon Energy in data sets ordered by magnitude.

Data set	Energy data
Ling *et al* (a) [16]	50 kvp^a^
Ling *et al* (b)[16]	280 kvp^a^
Koch *et al* [14]	662 keV^b^
Whillians and Hunt [15]	20 MV^c^

a X-ray tube peak kV: bremsstrahlung spectrum.

b Caesium 137 source—mono-energetic.

c Linac—bremsstrahlung spectrum.

**Table 4:** Best model fit estimates.

Data set	*R* ^2^	$\frac{\phi_{O}}{\phi_{D}}$	*φ*(mmHg^−1^)
Koch	0.99	1.69 ± 0.06	0.27 ± 0.02
Whillians and Hunt	0.92	1.62 ± 0.05	0.27 ± 0.03
Ling *et al* (a)	0.93	1.57 ± 0.05	0.23 ± 0.03
Ling *et al* (b)	0.98	2.01 ± 0.12	0.20 ± 0.04
Combined	0.94	1.63 ± 0.03	0.26 ± 0.02

**Table 5:** Thermal dependence of *φ*.

*T* (°C)	*φ*(mmHg^−1^)	$\frac{\varphi (T)}{\varphi (37{}^{\circ}C)}$
30	0.2783	1.08
32	0.2718	1.06
34	0.2656	1.03
36	0.2596	1.01
38	0.2538	0.99
40	0.2483	0.98

**Table 6:** Interaction probability with partial pressure.

Partial pressure	Percentage ionized DNA interacting with oxygen
0 mmHg	0%
0.5 mmHg	12.1%
1 mmHg	22.6%
1.5 mmHg	32%
2 mmHg	40.2%
2.5 mmHg	47.4%
3 mmHg	53.7%
10 mmHg	92.3%
20 mmHg	99.4%
100 mmHg	≈100%
